# Case of Hydrophobia

**Published:** 1848-04

**Authors:** Thomas T. Smiley


### *Case of Hydrophobia.* By Thomas T. Smiley, M. D.

*March 9th*, 1848.—Was called to visit John Henderson, aged 14 years. *Symptoms:* Mind perfectly clear and collected, eyes preternaturally bright, and hearing morbidly acute; complained of heat and constriction about the throat, and pain in the lumbar region. Pulse 80; small, and exhibiting very slight indications of febrile action. Tongue slightly furred, white and soft. Spasms about the muscles of the neck supervened every two or three minutes, and continued a few seconds. After each spasm a mouthful of white froth was ejected, having a tenacious appearance. Could swallow, by a strong effort, a portion of fluid taken into the mouth—a part also being violently ejected by the effort of deglutition. The difficulty of swallowing had been first observed on the morning of the 8th, after a good night's rest, and without any previous indications of indisposition. Had been bitten on the chin and under lip nine weeks previously by a dog exhibiting symptoms of rabies caninæ.

Both the history of the case and the unequivocal character of the symptoms pointing clearly to the nature of the disease, the mode of treatment became at once a matter of serious consideration. Reflecting on the acknowledged inefficacy of venesection, heretofore chiefly relied on for controlling the most prominent symptoms of hydrophobia; and no symptoms being present which on general principles required it, I determined not to resort to that method of treating the disease, but to make use of other remedies which had at least an equal chance of being useful, with less prospect of being injurious. For the purpose, therefore, of removing the muscular contractions and spasms of the glottis, I prescribed the following mixture: ℞. Tart. ant. et pot. gr. vi.; tinct. tolu, f℈i., aq. f℥vi., M. S. A table spoonful every five minutes till vomiting is produced; afterwards to be continued every hour. For the other most prominent symptom prescribed, the following as a liniment: ℞. Tinct. sapon. camph., tinct. canthar., liq. ammon. fff. P. E. M. S. To be well rubbed in along the whole course of the spine, by means of a soft piece of flannel, and to be repeated until the skin becomes abraded. No means being at hand for general bathing, ordered a hot mustard pediluvium.

*March* 10*th*, *at* 7, *A. M.*, the patient had passed a dreadful night, requiring the strength of several persons to hold him during the spasms, which had recurred frequently. The tart. ant. had all been taken without producing vomiting. The patient had complained of a little nausea, but the distressing symptoms did not appear to have been moderated, or in any way controlled by the exhibition of this medicine. Discontinued the further use of the tart. ant., and prescribed the following mixture: ℞. Spts. æth., sulph. comp., f℈ij.; tinct. theb., f℈j.; aq. camph., aq. cinnam., aq. pur., *aa* f℥ij. M. S. A tablespoonful every hour. Frictions to the spine to be continued as before directed.

At 11 o'clock, A. M., again visited the patient. He had succeeded in swallowing the mixture, as ordered at the previous visit, without much difficulty, and had had no spasms since he commenced taking it. Appears greatly relieved, and laughs and talks fluently. Sitting up on a stool by the fire. On entering the room had some difficulty in recognising the patient, his appearance was so much improved.

As the spts. æth. sulph. comp. appeared to have been evidently beneficial, and considering that, from the constitution of chloroform, it must possess analogous, if not identical properties, with a more powerful action on the nervous system, directed the mixture to be continued as before, and ordered ten drops of chloroform on a piece of sponge of suitable size, to be placed in the mouth, and inhaled by deep inspirations; to be repeated at intervals, more or less frequently, according to the exigency of the symptoms. Tongue moist, but heavily loaded, having a bilious appearance. Prescribed pulv. jal., gr. xxxvi.; hydrarg. chl. mit., gr. xij.; pulv. gambog., gr. vi.; mft. chart., No. 12, S. One every hour, till the bowels are freely disturbed.

*March* 11. *Morning.*—Had slept some during the previous night. Was sitting up on a chair before the fire, the floor around him covered with saliva: appears to be a genuine secretion from the salivary glands, being colourless and not frothy. Cathartic had operated slightly. Had been free from spasms during the night, while the medicines lasted; afterwards, more distressed. Articulation a little thick; mind collected. Conversed freely, and expressed himself greatly benefitted and relieved by the remedies prescribed. Asked to have another supply of the same, especially the "breathing mixture." Pulse 90; small, and less forcible than on the previous day. Directed the medicines to be continued as before.

*March* 12.—He had been nearly free from spasms during the previous day and night, and the difficulty of swallowing had greatly diminished. Had continued to take the mixture and inhale the chloroform, as directed. In the morning slept for an hour or two; was then suddenly roused by some person knocking violently at the door; complained of being disturbed, and expressed a desire to sleep again. Soon after told his mother to "bid the doctor good bye;" and expired quietly, without spasm or any violent effort.

REMARKS.

1. The spts. æth. sulph. comp.; tinct. theb. and chloroform having been prescribed with the view to produce a direct action on the symptoms accompanying the disease, the relative proportion and total amount of each exhibited during the treatment of the case, are worthy of being noted. Of the first and second, fʒvi. and fʒiij., respectively, were administered by the stomach; and of the chloroform, fʒij. by the lungs.

2. The first two, administered by the stomach, had prevented the recurrence of a paroxysm for three hours, before the chloroform was given by the lungs, and the combination of all three, when given as directed, prevented the recurrence of the most distressing symptoms during the subsequent progress of the case. Whether the first two would have continued to prevent the spasms, if given in the same or increased doses, would be difficult to determine.

3. It will be perceived that no attempt was made to give the chloroform to the extent of producing insensibility; nor was it considered desirable or proper to do so. The attempt to keep a a patient for hours or days constantly under the influence of that powerful remedy to such an extent, would itself in any case soon produce a fatal result; and the death of the patient might then be fairly referred to the remedy instead of to the disease. A constant but limited effect, just sufficient to prevent a recurrence of the spasms, was the object in view; and for this purpose small doses, frequently repeated, were directed.

4. The general result of the treatment in this case proves that the remedies used exerted a decided influence on the progress of the disease, and modified the symptoms to such an extent as greatly to relieve the distress, though it failed to cure. Possibly one step has been made in a course of treatment which may hereafter render hydrophobia a curable disease.

5. In the present case, circumstances did not admit of such a close and careful supervision as would have been desirable. The constant presence of a well qualified medical attendant, who could increase or diminish doses, and regulate the time of giving or withholding remedies, according to the immediate exigencies of the case, together with the use of all the collateral means necessary to give the fullest efficacy to the leading remedies, might have a great influence in producing a more favourable result.

6. It may be asked why the patient did not recover, if the spasms and other leading symptoms were under the control of remedies. It should be recollected that the disease is one thing—the symptoms are another. The spasm of the glottis and other phenomena attending the disease, are only symptoms, and not the disease itself. These symptoms are the manifestations of a morbid condition somewhere else—probably in one of the nervous centres—and may be modified or controlled without curing the morbid condition on which they depend.

